# Association between Programmed Cell Death 6 Interacting Protein Insertion/Deletion Polymorphism and the Risk of Breast Cancer in a Sample of Iranian Population

**DOI:** 10.1155/2015/854621

**Published:** 2015-05-03

**Authors:** Mohammad Hashemi, Javad Yousefi, Seyed Mehdi Hashemi, Shadi Amininia, Mahboubeh Ebrahimi, Mohsen Taheri, Saeid Ghavami

**Affiliations:** ^1^Cellular and Molecular Research Center, Zahedan University of Medical Sciences, Zahedan, Iran; ^2^Department of Clinical Biochemistry, School of Medicine, Zahedan University of Medical Sciences, Zahedan, Iran; ^3^Department of Internal Medicine, School of Medicine, Zahedan University of Medical Sciences, Zahedan, Iran; ^4^Genetic of Non-Communicable Disease Research Center, Zahedan University of Medical Sciences, Zahedan, Iran; ^5^Department of Human Anatomy and Cell Science, College of Medicine, Faculty of Health Sciences, University of Manitoba, Winnipeg, MB, Canada R3E 0J9; ^6^Manitoba Institute of Child Health, University of Manitoba, Winnipeg, MB, Canada R3E 0J9; ^7^Health Policy Research Center, Shiraz University of Medical Sciences, Shiraz, Iran

## Abstract

It has been suggested that genetic factors contribute to patients' vulnerability to breast cancer (BC). The programmed cell death 6 interacting protein (PDCD6IP) encodes for a protein that is known to bind to the products of the PDCD6 gene, which is involved in the apoptosis pathway. The aim of this case-control study is to investigate the relationship between the *PDCD6IP* 15 bp insertion/deletion (I/D) polymorphism (rs28381975) and BC risk in an Iranian population. A total of 491 females, including 266 BC patients and 225 control subjects without cancer, were enrolled into the study. Our findings revealed that the *PDCD6IP* 15 bp I/D polymorphism decreased the risk of BC in codominant (OR = 0.44, 95% CI = 0.31–0.65, *p* < 0.0001, I/D versus DD; OR = 0.39, 95% CI = 0.17–0.88, *p* = 0.030, I/I versus DD) and dominant (OR = 0.44, 95% CI = 0.30–0.63, *p* < 0.0001, D/I + I/I versus D/D) tested inheritance models. Also, the *PDCD6IP* I allele significantly decreased the risk of BC (OR = 0.59, 95% CI = 0.45–0.78, *p* < 0.001) compared to the D allele.

## 1. Introduction

Breast cancer (BC) is the most common cancer and the second leading cause of cancer death among women. It is recognized as an important health care problem worldwide, affecting more than 1 million women annually [1–3]. BC is one of the most frequent malignancies among Iranian women, and it comprises 21.4% of female cancers [4]. It has been proposed that BC affects Iranian women about a decade earlier than women of  western countries [5]. BC is known as a multifactor disease and its exact etiology is still unknown. We have previously investigated polymorphisms of human telomerase reverse transcriptase, cyclin E1, and lysosome-associated protein transmembrane 4 beta in BC and showed that genetic factors play important roles in the pathogenesis and progress of this malignancy [3, 6–8].

In humans, programmed cell death 6 interacting protein (PDCD6IP), which is also known as AIP1 (ALG2-interacting protein 1) and ALIX (ALG2-interacting protein 1), is mapped on chromosome 3p22.3. This gene in responsible for biosynthesis of a protein that is involved in protein transport and trafficking in the cells and it has several functions, including the abscission stage of cytokinesis, intraluminal endosomal vesicle formation, and enveloped virus budding [9]. In addition, the product of this gene binds to the product of the PDCD6 gene, a protein needed for apoptosis, in a calcium-dependent manner.

Apoptosis (programmed cell death I) is a tightly regulated pathway, which is involved in organism cell death [10–12]. Many factors play critical roles in apoptosis including, caspases [13, 14], pro- and antiapoptotic Bcl2 family proteins [15–17], and mitochondria proapoptotic proteins [18, 19]. Apoptosis imbalance has been reported to be critical in different cancers [14], and therefore many anticancer agents have been designed to target various apoptotic proteins/genes [20, 21].

Currently, little information is available regarding the association between* PDCD6IP* polymorphisms and cancer risk [9, 22]. To the best of our knowledge, there are no reports regarding the association between the 15 bp I/D variant within the* PDCD6IP* promoter and BC susceptibility. Therefore, in this study, we aimed to evaluate the impact of the* PDCD6IP* 15 bp I/D polymorphism on BC susceptibility in a sample of Iranian women.

## 2. Materials and Methods

### 2.1. Patients

This case-control study was performed in a southeast Iranian population, and 266 female BC patients and 225 female age-matched control subjects with no history of any types of cancer were enrolled into the study. Ethics approval for recruitment was obtained from the Local Ethics Committee of Zahedan University of Medical Sciences, and written informed consent was obtained from all patients and healthy individuals.

Blood samples were collected from patients and healthy controls, using EDTA-containing tubes, and DNA was extracted using the salting-out method, as described previously [23]. The quality of isolated DNA was assessed using electrophoresis on 1% agarose gel, quantitated spectrophotometrically, and stored at −20°C until further use.

### 2.2. Genotyping of 15 bp I/D PDCD6IP Polymorphism

**Figure 1:**
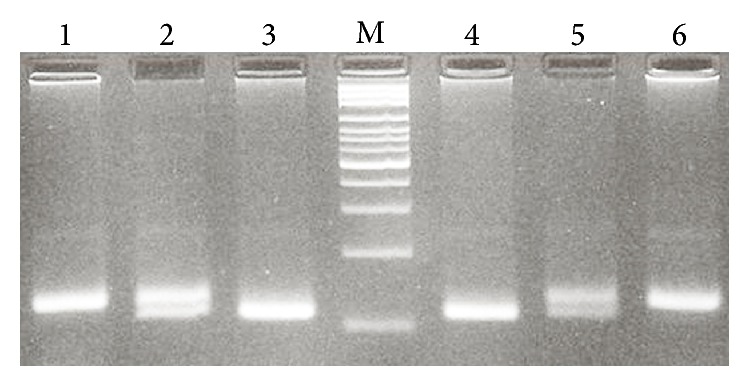
Photograph of the PDCD6IP 15 bp I/D (rs28381975) polymorphism PCR product. M: 100 bp DNA marker; lanes 1 and 6: I/I; lanes 2 and 5: I/D; lanes 3 and 4: D/D.

The polymerase chain reaction (PCR) was used for genotyping the 15 bp I/D PDCD6IP polymorphism using forward and reverse primer sequences as follows: 5′-ACCTGACAGTAAGCTGCACG-3′ and 5′-GGCAGTCCCAGGGTTATTGT-3′, respectively. PCR reactions consisted of a total volume of 20 *μ*L containing 250 *μ*M dNTPs, 0.5 *μ*M of each primer, 1.5 mM MgCl_2_, 1 U Taq DNA polymerase, and ~100 ng genomic DNA. The PCR cycling conditions consisted of an initial denaturing step for 5 min at 95°C followed by 30 cycles for 30 s at 95°C, 30 s at 60°C, and 30 s at 72°C, as well as a final extension step for 10 min at 72°C. The PCR products were visualized on a 3% agarose gel containing 0.5 *μ*g/mL of ethidium bromide and genotypes were determined (Figure 1). To certify genotyping quality, 20% of random samples were regenotyped and the results confirmed the previous genotyping outcomes (100% match).

### 2.3. Statistical Analysis

Statistical analysis was performed using the statistical package, SPSS 18 software. Demographic and biochemical parameters between the groups were analyzed using an independent sample *t*-test for continuous data and the *χ* ^2^ test for categorical data. The association between genotypes and BC was assessed by computing the odds ratio (OR) and 95% confidence intervals (95% CI) from logistic regression analyses. *p* < 0.05 was considered to be significant.

## 3. Results

The study group consisted of 266 female BC patients with an average age of 48.9 ± 11.1 years and 225 healthy females with a mean age of 50.0 ± 12.9 years. No significant difference in age was found between the groups (*p* = 0.306). The frequency distribution of the* PDCD6IP* 15 bp I/D genotypes in BC patients and control subjects is shown in Table 1. Our findings revealed that the* PDCD6IP* 15 bp I/D polymorphism decreased the risk of BC in codominant (OR = 0.44, 95% CI = 0.31–0.65, *p* < 0.0001, I/D versus DD; OR = 0.39, 95% CI = 0.17–0.88, *p* = 0.030, I/I versus DD) and dominant (OR = 0.44, 95% CI = 0.30–0.63, *p* < 0.0001, D/I + I/I versus D/D) tested inheritance models. In addition, the* PDCD6IP* I allele significantly decreased the risk of BC (OR = 0.59, 95% CI = 0.45–0.78, *p* < 0.001), compared to the D allele.

We also examined the impact of the* PDCD6IP* 15 bp I/D polymorphism and patients' clinicopathologic characteristics (Table 2). The results indicate that there is no significant association between this variant and age, pathological type, tumor size, grade, stage, estrogen receptor, progesterone receptor, and HER2 status (*p* > 0.05).

## 4. Discussion

In the present study, we investigated the impact of* PDCD6IP* 15 bp I/D on BC risk in a sample of Iranian women. Our results indicated that the* PDCD6IP* 15 bp I/D polymorphism decreased the risk of BC in codominant and dominant tested inheritance models. Similarly, the* PDCD6IP* I allele significantly decreased the risk of BC compared to the D allele. Moreover, we analyzed the association between the* PDCD6IP* 15 bp I/D polymorphism and clinicopathological characteristics of BC patients. The findings showed no significant association between the variant and clinicopathological characteristics.

To date, there have only been two reports that investigated the association between* PDCD6IP* 15 bp I/D and cancer risk. Yu et al. [22] evaluated the possible association between the* PDCD6IP* 15 bp I/D polymorphism and hepatocellular carcinoma (HCC). They found that subjects carrying I/D or I/I genotype had a significantly increased risk for HCC compared to individuals carrying the D/D genotype (OR = 1.39, 95% CI = 1.01–1.91, *p* = 0.033). The 15 bp insertion allele was associated with a 1.26-fold risk for HCC (95% CI = 1.04–1.54, *p* = 0.018). Liu et al. [9] investigated the association between the* PDCD6IP* 15 bp I/D polymorphism and non-small-cell lung cancer (NSCLC) in the Chinese Han population. They found that the* PDCD6IP* 15 bp I/D variant increased the risk NSCLC in a dominant inheritance model (OR = 1.72, 95% CI = 1.29–2.31, *p* < 0.01, ID + II versus DD). The* PDCD6IP* I allele was significantly associated with the risk of NSCLC (OR = 1.41, 95% CI = 1.18–1.69, *p* < 0.01). They also found that the* PDCD6IP* I/D polymorphism significantly increased the risk of advanced NSCLC [9]. It has been shown that the* PDCD6IP* rs1127732 C/T polymorphism is associated with BC [24]. Another genome-wide linkage analysis of bipolar disorder showed that the rs1127732 C/T variant increased the risk of bipolar disorder [25].

The* PDCD6IP* I allele has been shown to be associated with enhanced promoter activity [9]. Overexpression of PDCD6IP restores contact inhibition, promotes detachment-induced apoptosis, and reduces tumorigenicity in nude mice [26, 27]. Overexpression of an AIP1 deletion mutant protects HeLa and COS cells from apoptosis induced by serum starvation; thus, AIP1 might mediate, at least in part, the ALG-2 requirement for apoptosis [28]. Therefore, it can be concluded that* PDCD6IP* might be involved in regulation of apoptosis and that it might affect the balance between cell death and cell proliferation; thus,* PDCD6IP* could be involved in carcinogenesis. We showed that the* PDCD6IP* 15 bp I/D polymorphism decreased the risk of BC in codominant and dominant tested inheritance models, while the above two studies highlighted that the* PDCD6IP* 15 bp I/D polymorphism increases the risk of HCC and NSCLC in a Chinese population. Based on this controversy, we can conclude that the* PDCD6IP* gene product might have different effects on the regulation of apoptosis in different populations or that apoptosis might have different regulatory effects on cancer progress in different types of cancer.

It is well known that mutations lead to BC; however, other changes, such as chromosomal rearrangements, may lead to differential expression of oncogenes and tumor suppressor genes. Two BC susceptibility genes (BRCA1 and BRCA2) have been identified, and germline mutations in these genes are thought to account for between 5% and 10% of all BC cases [29]. Frameshift mutations as well as missense mutations are known to alter protein function. Defects in the biological action of the genome that are driven by various alterations, such as point mutations and chromosomal rearrangements, lead to the collapse of genome integrity, uncontrolled cell proliferation, and failure of apoptotic cell death [30].

A limitation of this study is that we did not use the association between several genes together and the BC outcome in the statistical analysis; this may cause different results.

In conclusion, our findings indicate that the* PDCD6IP* 15 bp I/D polymorphism decreases the risk of BC in an Iranian population. Further studies with different ethnicities are required to confirm our findings.

## Figures and Tables

**Table 1:** The genotypes and allele distribution of PCD6IP 15 bp I/D (rs28381975) variants in breast cancer (BC) patients and the control group.

PCD6IP	Patients	Control group	OR (95% CI)	*p* value
	*n* (%)	*n* (%)		
Codominant				
D/D	139 (52.3)	73 (32.4)	1.00	—
I/D	116 (43.6)	137 (60.9)	0.44 (0.31–0.65)	<0.0001
I/I	11 (4.1)	15 (6.7)	0.39 (0.17–0.88)	0.030
Dominant				
D/D	139 (52.3)	73 (32.4)	1.00	—
D/I + I/I	127 (47.7)	152 (67.6)	0.44 (0.30–0.63)	<0.0001
Recessive				
D/D + D/I	256 (95.9)	210 (93.3)	1.00	—
I/I	11 (4.1)	15 (6.7)	0.60 (0.27–1.34)	0.229
Allele				
D	394 (74.1)	283 (62.9)	1.00	—
I	138 (25.9)	167 (37.1)	0.59 (0.45–0.78)	<0.001

**Table 2:** Association between the *PDCD6IP *15 bp I/D polymorphism and clinicopathological characteristics.

Variables	*PDCD6IP* 15 bp I/D			*p* value
	D/D	I/D	I/I	
Age (years)				0.757
≤50	75	66	7	
>50	61	46	4	
Pathological type				0.909
Ductal	83	63	6	
Others	42	34	4	
Tumor size (cm)				0.111
≤2	55	31	2	
>2	72	66	8	
TNM stage				0.388
I	33	18	0	
II	50	38	5	
III	35	31	3	
IV	17	18	3	
Grade				0.989
I	27	20	2	
II	71	47	6	
III + IV	18	13	1	
Estrogen receptor				0.625
Positive	69	54	5	
Negative	43	42	5	
Progesterone receptor				0.460
Positive	63	56	7	
Negative	48	38	2	
HER2				0.141
Positive	76	47	6	
Negative	57	59	5	
